# Bis[μ-bis­(pyridin-2-yl)methanone oxime-κ^3^*N*:,*N*′,*N*′′]bis­[di­acetato-κ^2^*O*,*O*′;κ*O*-zinc(II)]

**DOI:** 10.1107/S2414314624001226

**Published:** 2024-02-16

**Authors:** Guy Crundwell, Nigel E. Crundwell, Barry L. Westcott

**Affiliations:** a Central Connecticut State University, Department of Chemistry & Biochemistry 1619 Stanley Street New Britain CT 06053 USA; Purdue University, USA

**Keywords:** crystal structure, dpko, zinc

## Abstract

The structure of the title complex, [Zn_2_(C_2_H_3_O_2_)_4_(C_11_H_9_N_3_O)_2_], is triclinic containing half of the mol­ecule in the asymmetric unit. Each zinc atom is coordinated to a pyridyl and oxime nitro­gen from one di-2-pyridyl ketone oxime (dpko) ligand and a third nitro­gen from the other dpko pyridyl ring. Additionally, each zinc is coordinated to two acetato anions, one of which is bidentate and the other monodentate. The uncoordinated oxygen of the monodentate acetato group is involved in a hydrogen bond with the oxime hydrogen. The packing in the crystal is assisted by weak C—H⋯O inter­actions between acetato groups and neighboring pyridyl rings.

## Abstract 2

The structure of the title compound is triclinic containing half of the mol­ecule in the asymmetric unit. Each zinc atom is coordinated to a pyridyl and oxime nitro­gen from one ligand and a third nitro­gen from the other dpko pyridyl ring and two acetato anions.

## Graphical abstract

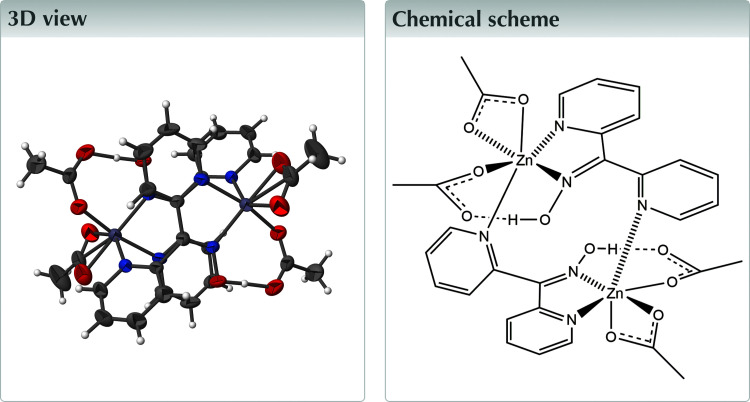

## Structure description

The three N atoms in dpko can act as ligands in a variety of ways. Previous reactions of Zn^II^ with dpko led to mol­ecules of the form Zn(dpko)Cl_2_ (Alexiou *et al.*, 2003 ▸; Gökce *et al.*, 2019 ▸) and Zn(dpko)Br_2_ (Westcott *et al.*, 2016 ▸) where both pyridyl N atoms are bonding to the metal and the oxime group is directed away from the metal center. Dpko ligands with zinc have also been shown to retain their bidentate nature, yet they opt to bond *via* one pyridyl nitro­gen and the oxime nitro­gen (Tarushi *et al.*, 2013 ▸). Finally, in this complex a third motif is seen; one where a pyridyl nitro­gen and oxime nitro­gen bond to one zinc and the other pyridyl nitro­gen binds to another. In this case a dimer is made and is analogous to Cu^2+^ complexes with dpko (Goher & Mautner, 1999 ▸) and to Mg^2+^ complexes with dpko (Milios *et al.*, 2005 ▸).

**Figure 1:**
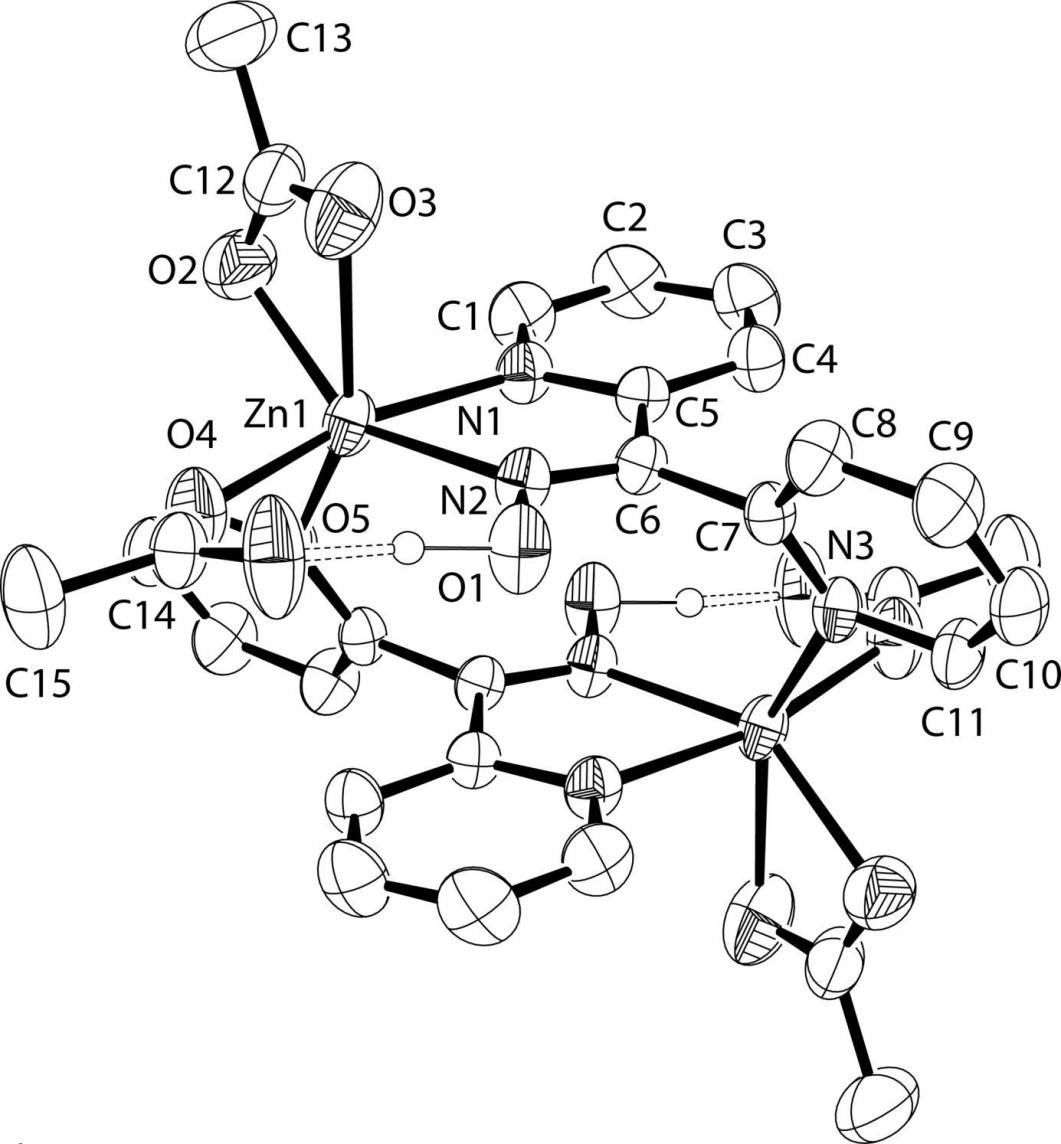
An *ORTEP* style (Farrugia, 2012 ▸) view of the title compound. Displace­ment ellipsoids are drawn at the 50% probability level. All hydrogen atoms not involved in hydrogen bonding have been omitted and non-H atoms generated by the inversion center have not been labeled.

The asymmetric-unit of the the title complex, Fig. 1 ▸, comprises one-half molecule with the full molecule generated by inversion symmetry. Two acetate anions are also coordin­ated to the zinc. The first acetato group bonds with both O atoms at bond lengths of 2.1369 (17) and 2.289 (2) Å and the second acetato group coordinates through one oxygen at 2.0513 (14) Å. The second oxygen on the monodentate acetate is hydrogen bonded to the hydrogen on the oxime, Table 1 ▸. The packing in the crystal is assisted by weak C—H⋯O inter­actions between acetato groups and neighboring pyridyl rings(Table 1 ▸).

## Synthesis and crystallization

Zinc acetate dihydrate and di-2-pyridyl ketone oxime (dpko) were used as received from Mallinckrodt and Sigma-Aldrich, respectively. A 15 ml solution of 0.3474 g (1.58 mmol) of zinc acetate dihydrate in aceto­nitrile was combined with a 15 ml aceto­nitrile solution of 0.3227 g (1.62 mmol) of dpko and stirred for 10 minutes, producing a colorless solution. Diffraction-quality, colorless crystals formed *via* slow evaporation of solvent within 24 h. Crystals were harvested from the evaporating solutions and decompose upon heating. IR (cm^−1^) 1960(*wb*), 1710(*mb*), 1590(*s*), 1560(*s*), 1480(*m*), 1420(*s*), 1300(*w*), 1210(*w*), 1110(*w*), 1080(*sb*), 1010(*s*), 789(*s*), 754(*m*), 698(*m*), 675(*m*), 659(*s*).

## Refinement

Crystal data, data collection and structure refinement details are summarized in Table 2 ▸.

## Supplementary Material

Crystal structure: contains datablock(s) I. DOI: 10.1107/S2414314624001226/zl4065sup1.cif

Structure factors: contains datablock(s) I. DOI: 10.1107/S2414314624001226/zl4065Isup2.hkl

CCDC reference: 2331143

Additional supporting information:  crystallographic information; 3D view; checkCIF report

## Figures and Tables

**Table 1:** Hydrogen-bond geometry (Å, °)

*D*—H⋯*A*	*D*—H	H⋯*A*	*D*⋯*A*	*D*—H⋯*A*
O1—H1⋯O5	1.11 (4)	1.32 (4)	2.428 (2)	176 (3)
C2—H2⋯O1^ii^	0.93	2.34	3.245 (2)	164
C3—H3⋯O3^iii^	0.93	2.58	3.293 (3)	134
C9—H9⋯03^iv^	0.93	2.59	3.361 (3)	141
C11—H11⋯O2^i^	0.93	2.38	2.941 (3)	119

Symmetry codes: (i) 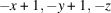; (ii) 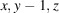; (iii) 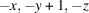; (iv) 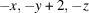.

**Table 2:** Experimental details

Crystal data	
Chemical formula	[Zn_2_(C_2_H_3_O_2_)_4_(C_11_H_9_N_3_O)_2_]
*M*_r_	765.34
Crystal system, space group	Triclinic, *P* 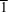
Temperature (K)	293
*a*, *b*, *c* (Å)	8.3549 (7), 9.3366 (8), 12.3971 (7)
α, β, γ (°)	69.409 (7), 75.524 (6), 65.217 (8)
*V* (Å^3^)	815.88 (13)
*Z*	1
Radiation type	Mo *K*α
μ (mm^−1^)	1.54
Crystal size (mm)	0.35 × 0.32 × 0.31

Data collection	
Diffractometer	Xcalibur, Sapphire3
Absorption correction	Multi-scan (*CrysAlis PRO*; Rigaku OD, 2019 ▸)
*T*_min_, *T*_max_	0.907, 1.000
No. of measured, independent and observed [*I* > 2σ(*I*)] reflections	10420, 5737, 4546
*R*_int_	0.022
(sin θ/λ)_max_ (Å^−1^)	0.778

Refinement	
*R*[*F*^2^ > 2σ(*F*^2^)], *wR*(*F*^2^), *S*	0.039, 0.098, 1.03
No. of reflections	5737
No. of parameters	223
H-atom treatment	H atoms treated by a mixture of independent and constrained refinement
Δρ_max_, Δρ_min_ (e Å^−3^)	0.66, −0.22

Computer programs: *CrysAlis PRO* (Rigaku OD, 2019 ▸), *SHELXS* (Sheldrick, 2008 ▸), *SHELXL* (Sheldrick, 2015 ▸), and *OLEX2* (Dolomanov *et al.*, 2009 ▸).

## full crystallographic data

### Bis[µ-bis(pyridin-2-yl)methanone oxime-κ3N:N',N'']bis[diacetato-κ2O,O';κO-zinc(II)] Crystal data

**Table:**

[Zn_2_(C_2_H_3_O_2_)_4_(C_11_H_9_N_3_O)_2_]	*Z* = 1
*M**_r_* = 765.34	*F*(000) = 392
Triclinic, *P*1	*D*_x_ = 1.558 Mg m^−^^3^
*a* = 8.3549 (7) Å	Mo *K*α radiation, λ = 0.71073 Å
*b* = 9.3366 (8) Å	Cell parameters from 4170 reflections
*c* = 12.3971 (7) Å	θ = 4.5–33.0°
α = 69.409 (7)°	µ = 1.54 mm^−^^1^
β = 75.524 (6)°	*T* = 293 K
γ = 65.217 (8)°	Block, colorless
*V* = 815.88 (13) Å^3^	0.35 × 0.32 × 0.31 mm

### Bis[µ-bis(pyridin-2-yl)methanone oxime-κ3N:N',N'']bis[diacetato-κ2O,O';κO-zinc(II)] Data collection

**Table:**

Xcalibur, Sapphire3 diffractometer	5737 independent reflections
Radiation source: Enhance (Mo) X-ray Source	4546 reflections with *I* > 2σ(*I*)
Graphite monochromator	*R*_int_ = 0.022
Detector resolution: 16.1790 pixels mm^-1^	θ_max_ = 33.6°, θ_min_ = 4.2°
ω scans	*h* = −11→12
Absorption correction: multi-scan (CrysAlisPro; Rigaku OD, 2019)	*k* = −13→13
*T*_min_ = 0.907, *T*_max_ = 1.000	*l* = −18→18
10420 measured reflections	

### Bis[µ-bis(pyridin-2-yl)methanone oxime-κ3N:N',N'']bis[diacetato-κ2O,O';κO-zinc(II)] Refinement

**Table:**

Refinement on *F*^2^	Primary atom site location: iterative
Least-squares matrix: full	Hydrogen site location: mixed
*R*[*F*^2^ > 2σ(*F*^2^)] = 0.039	H atoms treated by a mixture of independent and constrained refinement
*wR*(*F*^2^) = 0.098	*w* = 1/[σ^2^(*F*_o_^2^) + (0.0443*P*)^2^ + 0.1935*P*] where *P* = (*F*_o_^2^ + 2*F*_c_^2^)/3
*S* = 1.03	(Δ/σ)_max_ < 0.001
5737 reflections	Δρ_max_ = 0.66 e Å^−^^3^
223 parameters	Δρ_min_ = −0.22 e Å^−^^3^
0 restraints	

### Bis[µ-bis(pyridin-2-yl)methanone oxime-κ3N:N',N'']bis[diacetato-κ2O,O';κO-zinc(II)] Special details

**Table:**

Geometry. All esds (except the esd in the dihedral angle between two l.s. planes) are estimated using the full covariance matrix. The cell esds are taken into account individually in the estimation of esds in distances, angles and torsion angles; correlations between esds in cell parameters are only used when they are defined by crystal symmetry. An approximate (isotropic) treatment of cell esds is used for estimating esds involving l.s. planes.
Refinement. Hydrogen atoms on sp2 and sp3 carbons were placed at calculated positions with a C—H distance of 0.93?Å and 0.96?Å and were included in the refinement in riding motion approximation with Uiso = 1.2Ueq or 1.5Ueq of the carrier atom, respectively. The position and thermal parameters for the oxime hydrogen were allowed to refine freely.

### Bis[µ-bis(pyridin-2-yl)methanone oxime-κ3N:N',N'']bis[diacetato-κ2O,O';κO-zinc(II)] Fractional atomic coordinates and isotropic or equivalent isotropic displacement parameters (Å^2^)

**Table:**

	*x*	*y*	*z*	*U*_iso_*/*U*_eq_	
Zn1	0.34622 (3)	0.46689 (2)	0.21836 (2)	0.03562 (8)	
O1	0.3171 (2)	0.81600 (16)	0.04557 (12)	0.0489 (4)	
O2	0.2284 (2)	0.3158 (2)	0.35870 (14)	0.0625 (4)	
O3	0.0490 (3)	0.5591 (2)	0.28225 (15)	0.0738 (5)	
O4	0.4272 (2)	0.56182 (17)	0.31195 (12)	0.0517 (4)	
O5	0.3442 (3)	0.83015 (19)	0.23171 (15)	0.0721 (6)	
N1	0.3011 (2)	0.38026 (17)	0.08947 (13)	0.0362 (3)	
N2	0.3064 (2)	0.66897 (16)	0.06028 (12)	0.0338 (3)	
N3	0.3964 (2)	0.73732 (16)	−0.23140 (12)	0.0327 (3)	
C1	0.2935 (3)	0.2329 (2)	0.10808 (19)	0.0475 (5)	
H1A	0.318212	0.155161	0.179385	0.057*	
C2	0.2505 (3)	0.1924 (2)	0.0253 (2)	0.0549 (6)	
H2	0.247728	0.088449	0.040771	0.066*	
C3	0.2119 (3)	0.3053 (3)	−0.0797 (2)	0.0523 (5)	
H3	0.180763	0.280147	−0.135924	0.063*	
C4	0.2202 (3)	0.4589 (2)	−0.10074 (17)	0.0410 (4)	
H4	0.194267	0.538517	−0.171163	0.049*	
C5	0.2675 (2)	0.49042 (19)	−0.01513 (14)	0.0314 (3)	
C6	0.2823 (2)	0.64842 (18)	−0.03036 (13)	0.0300 (3)	
C7	0.2681 (2)	0.77575 (19)	−0.14482 (14)	0.0310 (3)	
C8	0.1286 (3)	0.9258 (2)	−0.15985 (17)	0.0434 (4)	
H8	0.042857	0.949951	−0.097911	0.052*	
C9	0.1179 (3)	1.0396 (2)	−0.26807 (19)	0.0523 (5)	
H9	0.025274	1.141321	−0.279936	0.063*	
C10	0.2468 (3)	0.9997 (2)	−0.35777 (18)	0.0489 (5)	
H10	0.241831	1.073160	−0.431699	0.059*	
C11	0.3830 (3)	0.8493 (2)	−0.33611 (15)	0.0419 (4)	
H11	0.470518	0.823590	−0.396915	0.050*	
C12	0.0764 (3)	0.4213 (3)	0.35166 (18)	0.0533 (5)	
C13	−0.0764 (4)	0.3756 (5)	0.4298 (3)	0.0938 (12)	
H13A	−0.181804	0.435492	0.391611	0.141*	
H13B	−0.096138	0.402581	0.501117	0.141*	
H13C	−0.047996	0.259790	0.446085	0.141*	
C14	0.4072 (3)	0.7035 (2)	0.30937 (16)	0.0426 (4)	
C15	0.4641 (4)	0.7266 (3)	0.4055 (2)	0.0684 (7)	
H15A	0.461453	0.638302	0.474543	0.103*	
H15B	0.384817	0.829313	0.420185	0.103*	
H15C	0.582636	0.726925	0.383512	0.103*	
H1	0.325 (5)	0.821 (4)	0.132 (3)	0.116 (13)*	

### Bis[µ-bis(pyridin-2-yl)methanone oxime-κ3N:N',N'']bis[diacetato-κ2O,O';κO-zinc(II)] Atomic displacement parameters (Å^2^)

**Table:**

	*U*^11^	*U*^22^	*U*^33^	*U*^12^	*U*^13^	*U*^23^
Zn1	0.04561 (13)	0.02653 (10)	0.02801 (10)	−0.00898 (8)	−0.00571 (8)	−0.00425 (7)
O1	0.0854 (11)	0.0274 (6)	0.0412 (7)	−0.0243 (7)	−0.0195 (7)	−0.0054 (5)
O2	0.0476 (9)	0.0701 (11)	0.0524 (9)	−0.0126 (8)	−0.0018 (7)	−0.0110 (8)
O3	0.0920 (14)	0.0576 (11)	0.0510 (10)	−0.0185 (10)	−0.0065 (9)	−0.0037 (8)
O4	0.0768 (11)	0.0382 (7)	0.0431 (7)	−0.0183 (7)	−0.0202 (7)	−0.0095 (6)
O5	0.1310 (17)	0.0374 (8)	0.0516 (9)	−0.0177 (9)	−0.0432 (10)	−0.0107 (7)
N1	0.0459 (8)	0.0255 (6)	0.0353 (7)	−0.0141 (6)	−0.0083 (6)	−0.0028 (5)
N2	0.0482 (8)	0.0231 (6)	0.0302 (6)	−0.0126 (6)	−0.0065 (6)	−0.0068 (5)
N3	0.0411 (8)	0.0261 (6)	0.0263 (6)	−0.0082 (6)	−0.0082 (5)	−0.0043 (5)
C1	0.0612 (12)	0.0276 (8)	0.0519 (11)	−0.0198 (8)	−0.0135 (10)	0.0001 (7)
C2	0.0700 (15)	0.0314 (9)	0.0726 (15)	−0.0253 (10)	−0.0184 (12)	−0.0104 (9)
C3	0.0655 (14)	0.0443 (11)	0.0614 (13)	−0.0246 (10)	−0.0176 (11)	−0.0188 (9)
C4	0.0533 (11)	0.0351 (9)	0.0398 (9)	−0.0190 (8)	−0.0126 (8)	−0.0083 (7)
C5	0.0364 (8)	0.0251 (7)	0.0319 (7)	−0.0107 (6)	−0.0055 (6)	−0.0070 (6)
C6	0.0360 (8)	0.0231 (7)	0.0281 (7)	−0.0092 (6)	−0.0046 (6)	−0.0052 (5)
C7	0.0396 (8)	0.0241 (7)	0.0292 (7)	−0.0109 (6)	−0.0097 (6)	−0.0046 (5)
C8	0.0458 (10)	0.0310 (8)	0.0399 (9)	−0.0036 (7)	−0.0058 (8)	−0.0069 (7)
C9	0.0523 (12)	0.0321 (9)	0.0515 (12)	−0.0005 (8)	−0.0151 (9)	0.0004 (8)
C10	0.0546 (12)	0.0396 (10)	0.0379 (9)	−0.0120 (9)	−0.0167 (9)	0.0073 (7)
C11	0.0472 (10)	0.0395 (9)	0.0291 (8)	−0.0110 (8)	−0.0084 (7)	−0.0014 (7)
C12	0.0519 (12)	0.0612 (13)	0.0353 (9)	−0.0152 (10)	−0.0035 (8)	−0.0081 (9)
C13	0.0545 (16)	0.121 (3)	0.0676 (18)	−0.0295 (18)	0.0021 (13)	0.0081 (17)
C14	0.0539 (11)	0.0412 (9)	0.0349 (9)	−0.0148 (8)	−0.0078 (8)	−0.0149 (7)
C15	0.107 (2)	0.0603 (14)	0.0537 (13)	−0.0356 (15)	−0.0343 (14)	−0.0118 (11)

### Bis[µ-bis(pyridin-2-yl)methanone oxime-κ3N:N',N'']bis[diacetato-κ2O,O';κO-zinc(II)] Geometric parameters (Å, º)

**Table:**

Zn1—O2	2.1369 (17)	C3—H3	0.9300
Zn1—O3	2.289 (2)	C3—C4	1.393 (3)
Zn1—O4	2.0513 (14)	C4—H4	0.9300
Zn1—N1	2.1944 (16)	C4—C5	1.377 (3)
Zn1—N2	2.1702 (14)	C5—C6	1.474 (2)
Zn1—N3^i^	2.1921 (14)	C6—C7	1.490 (2)
Zn1—C12	2.538 (2)	C7—C8	1.383 (2)
O1—N2	1.3581 (18)	C8—H8	0.9300
O1—H1	1.11 (4)	C8—C9	1.384 (3)
O2—C12	1.240 (3)	C9—H9	0.9300
O3—C12	1.233 (3)	C9—C10	1.375 (3)
O4—C14	1.251 (2)	C10—H10	0.9300
O5—C14	1.238 (2)	C10—C11	1.374 (3)
O5—H1	1.32 (4)	C11—H11	0.9300
N1—C1	1.339 (2)	C12—C13	1.516 (4)
N1—C5	1.349 (2)	C13—H13A	0.9600
N2—C6	1.279 (2)	C13—H13B	0.9600
N3—C7	1.343 (2)	C13—H13C	0.9600
N3—C11	1.346 (2)	C14—C15	1.498 (3)
C1—H1A	0.9300	C15—H15A	0.9600
C1—C2	1.377 (3)	C15—H15B	0.9600
C2—H2	0.9300	C15—H15C	0.9600
C2—C3	1.368 (3)		
			
O2—Zn1—O3	58.16 (6)	C5—C4—C3	118.68 (18)
O2—Zn1—N1	92.26 (6)	C5—C4—H4	120.7
O2—Zn1—N2	146.35 (6)	N1—C5—C4	122.62 (15)
O2—Zn1—N3^i^	89.78 (6)	N1—C5—C6	114.95 (14)
O2—Zn1—C12	29.18 (7)	C4—C5—C6	122.42 (15)
O3—Zn1—C12	29.01 (7)	N2—C6—C5	115.96 (14)
O4—Zn1—O2	99.02 (7)	N2—C6—C7	122.71 (14)
O4—Zn1—O3	98.14 (7)	C5—C6—C7	121.32 (14)
O4—Zn1—N1	168.00 (6)	N3—C7—C6	116.84 (14)
O4—Zn1—N2	98.84 (6)	N3—C7—C8	122.22 (15)
O4—Zn1—N3^i^	88.29 (6)	C8—C7—C6	120.94 (16)
O4—Zn1—C12	100.79 (7)	C7—C8—H8	120.4
N1—Zn1—O3	91.27 (7)	C7—C8—C9	119.26 (18)
N1—Zn1—C12	91.05 (7)	C9—C8—H8	120.4
N2—Zn1—O3	91.18 (6)	C8—C9—H9	120.6
N2—Zn1—N1	73.40 (5)	C10—C9—C8	118.83 (18)
N2—Zn1—N3^i^	119.03 (6)	C10—C9—H9	120.6
N2—Zn1—C12	118.85 (7)	C9—C10—H10	120.6
N3^i^—Zn1—O3	147.87 (6)	C11—C10—C9	118.74 (17)
N3^i^—Zn1—N1	87.66 (6)	C11—C10—H10	120.6
N3^i^—Zn1—C12	118.87 (6)	N3—C11—C10	123.36 (18)
N2—O1—H1	107.4 (19)	N3—C11—H11	118.3
C12—O2—Zn1	93.65 (15)	C10—C11—H11	118.3
C12—O3—Zn1	86.75 (16)	O2—C12—Zn1	57.18 (12)
C14—O4—Zn1	134.88 (14)	O2—C12—C13	118.1 (2)
C14—O5—H1	120.5 (16)	O3—C12—Zn1	64.23 (14)
C1—N1—Zn1	125.79 (13)	O3—C12—O2	121.3 (2)
C1—N1—C5	117.95 (16)	O3—C12—C13	120.6 (2)
C5—N1—Zn1	116.13 (11)	C13—C12—Zn1	173.8 (2)
O1—N2—Zn1	125.25 (11)	C12—C13—H13A	109.5
C6—N2—Zn1	119.18 (11)	C12—C13—H13B	109.5
C6—N2—O1	115.40 (13)	C12—C13—H13C	109.5
C7—N3—Zn1^i^	127.73 (11)	H13A—C13—H13B	109.5
C7—N3—C11	117.57 (15)	H13A—C13—H13C	109.5
C11—N3—Zn1^i^	113.67 (12)	H13B—C13—H13C	109.5
N1—C1—H1A	118.9	O4—C14—C15	119.21 (19)
N1—C1—C2	122.28 (18)	O5—C14—O4	125.11 (18)
C2—C1—H1A	118.9	O5—C14—C15	115.68 (19)
C1—C2—H2	120.1	C14—C15—H15A	109.5
C3—C2—C1	119.88 (18)	C14—C15—H15B	109.5
C3—C2—H2	120.1	C14—C15—H15C	109.5
C2—C3—H3	120.7	H15A—C15—H15B	109.5
C2—C3—C4	118.55 (19)	H15A—C15—H15C	109.5
C4—C3—H3	120.7	H15B—C15—H15C	109.5
C3—C4—H4	120.7		
			
Zn1—O2—C12—O3	−3.7 (3)	N2—C6—C7—C8	−64.7 (3)
Zn1—O2—C12—C13	175.4 (2)	N3—C7—C8—C9	1.3 (3)
Zn1—O3—C12—O2	3.4 (3)	C1—N1—C5—C4	2.3 (3)
Zn1—O3—C12—C13	−175.6 (3)	C1—N1—C5—C6	−178.61 (17)
Zn1—O4—C14—O5	−10.1 (4)	C1—C2—C3—C4	1.0 (4)
Zn1—O4—C14—C15	170.56 (18)	C2—C3—C4—C5	0.2 (3)
Zn1—N1—C1—C2	174.66 (17)	C3—C4—C5—N1	−2.0 (3)
Zn1—N1—C5—C4	−173.72 (14)	C3—C4—C5—C6	179.04 (19)
Zn1—N1—C5—C6	5.3 (2)	C4—C5—C6—N2	171.92 (17)
Zn1—N2—C6—C5	5.5 (2)	C4—C5—C6—C7	−7.5 (3)
Zn1—N2—C6—C7	−175.07 (12)	C5—N1—C1—C2	−1.0 (3)
Zn1^i^—N3—C7—C6	−14.1 (2)	C5—C6—C7—N3	−65.3 (2)
Zn1^i^—N3—C7—C8	165.91 (14)	C5—C6—C7—C8	114.7 (2)
Zn1^i^—N3—C11—C10	−168.65 (17)	C6—C7—C8—C9	−178.66 (19)
O1—N2—C6—C5	−179.02 (15)	C7—N3—C11—C10	0.6 (3)
O1—N2—C6—C7	0.4 (3)	C7—C8—C9—C10	0.1 (3)
N1—C1—C2—C3	−0.7 (4)	C8—C9—C10—C11	−1.1 (4)
N1—C5—C6—N2	−7.1 (2)	C9—C10—C11—N3	0.7 (3)
N1—C5—C6—C7	173.44 (16)	C11—N3—C7—C6	178.31 (16)
N2—C6—C7—N3	115.34 (19)	C11—N3—C7—C8	−1.6 (3)

Symmetry code: (i) −*x*+1, −*y*+1, −*z*.

### Bis[µ-bis(pyridin-2-yl)methanone oxime-κ3N:N',N'']bis[diacetato-κ2O,O';κO-zinc(II)] Hydrogen-bond geometry (Å, º)

**Table:**

*D*—H···*A*	*D*—H	H···*A*	*D*···*A*	*D*—H···*A*
O1—H1···O5	1.11 (4)	1.32 (4)	2.428 (2)	176 (3)
C2—H2···O1^ii^	0.93	2.34	3.245 (2)	164
C3—H3···O3^iii^	0.93	2.58	3.293 (3)	134
C9—H9···03^iv^	0.93	2.59	3.361 (3)	141
C11—H11···O2^i^	0.93	2.38	2.941 (3)	119

Symmetry codes: (i) −*x*+1, −*y*+1, −*z*; (ii) *x*, *y*−1, *z*; (iii) −*x*, −*y*+1, −*z*; (iv) −*x*, −*y*+2, −*z*.
